# The Role of Physical Activity in Moderating Psychopathological Symptoms and Quality of Life Among Adult Cancer Survivors: A Cross-Sectional Study

**DOI:** 10.3390/healthcare13172232

**Published:** 2025-09-06

**Authors:** Andreia Pereira Tavares, Paula Saraiva Carvalho, Ana Torres

**Affiliations:** 1 Department of Psychology and Education, University of Beira Interior, 6200-209 Covilhã, Portugal; psc@ubi.pt (P.S.C.); ana.carla.torres@ubi.pt (A.T.); 2 Research Center in Sports Sciences, Health Sciences and Human Development (CIDESD), University of Beira Interior, 6201-001 Covilhã, Portugal; 3 RISE-Health, Department of Psychology and Education, Faculty of Human and Social Sciences, University of Beira Interior, 6200-209 Covilhã, Portugal

**Keywords:** cancer survivors, physical activity, depression, anxiety, quality of life, integrated healthcare

## Abstract

**Background/Objectives**: Several studies indicate that physical activity is both safe and beneficial for most cancer survivors—before, during and after treatment. These benefits include improved mental health and a subsequent positive impact on quality of life. This study aimed to (1) assess the mental health of cancer survivors in terms of depression and anxiety, (2) analyze levels of physical activity within the sample, and (3) explore the relationship between psychopathological symptoms, physical activity, and perceived quality of life. **Methods**: This is a cross-sectional study of 55 cancer survivors, with a mean age of 62.27 ± 11.91, living in inland of Portugal and not undergoing palliative care. Data were collected using a sociodemographic, clinical and physical activity questionnaire, the Patient Health Questionnaire (PHQ-9), the Generalized Anxiety Disorder scale (GAD-7), the European Organization for Research and Treatment of Cancer Quality of Life Questionnaire Core-30 (EORTC QLQ-C30), and the Godin Leisure-Time Exercise Questionnaire (GLTEQ). Data was analyzed using descriptive statistics, Cronbach’s coefficient to assess the internal consistency, Spearman’s correlation, and multiple linear regression. **Results**: The results revealed significant associations between physical activity, psychopathological symptomatology, and quality of life. Specifically, the interaction between depression and physical activity had a negative impact on quality of life (B = −0.181; 95% CI −0.291 to −0.070; *p* = 0.002), whereas the interaction between anxiety and physical activity showed a positive effect (B = 0.165; 95% CI 0.037 to 0.293; *p* = 0.013). **Conclusions**: Physical activity enhances the quality of life of cancer survivors and moderates the negative impact of psychopathological symptoms. This highlights the importance of promoting healthy lifestyles and empowering healthcare professionals to recommend supervised physical activity as part of integrated and personalized care. Further studies should explore the relationship between other psychopathological symptoms, such as somatization, and physical activity in relation to quality of life.

## 1. Introduction

In recent years, surviving cancer has become a reality for many cancer patients, as cancer-related mortality is decreasing, and both the prognosis and course of the disease have improved significantly due to progress in cancer screening and early detection, as well as enhanced diagnostic, treatment and support services [1,2,3]. According to the International Agency for Research on Cancer, in 2022, approximately 53.5 million patients would be alive five years after their cancer diagnosis [4].

There is no consensus in the literature on a precise and clear definition of cancer survivor. Nevertheless, the National Coalition for Cancer Survivorship [5] has broadly defined cancer survivorship as a continuous experience, from diagnosis to the end of life. Furthermore, this concept considers the impact of the diagnosis on friends, caregivers, and family.

Cancer survivors are a vulnerable group, exposed to multiple challenges that can compromise their physical and psychological well-being and quality of life. These include feelings of isolation and loneliness [6], fear related to recurrence, relapse or progression of cancer [7], uncertainty about the future [8], persistent fatigue [9,10], chronic pain [9], and psychopathological symptoms such as depression and anxiety [11]. Therefore, assessing the quality of life and psychological well-being of cancer survivors is an essential part of understanding survival and the long-term effects of cancer and its treatments [12,13].

According to the National Cancer Institute [14], in 2022, approximately 36.7% of cancer survivors aged 18 or older reported not engaging in any physical activities during their leisure time. Previous studies have shown that physical activity is safe and beneficial practices for most individuals before, during and after cancer treatment, improving quality of life and increasing energy to engage in daily life and leisure activities [10,15,16,17]. Moreover, it contributes to coping with both side and late effects of treatment, enhances longevity and survival, and may even decrease the chances of developing a new type of cancer [10,18].

Engaging in physical activity according to medical recommendations can also improve cancer-related outcomes, including mental health, by reducing symptoms of depression and anxiety [10,19,20]. A systematic review and meta-analyses involving 1228 participants conducted by Sun et al. [19] revealed that engaging in physical activity can significantly decrease anxiety levels in breast cancer survivors, as well as an effect on depression levels. Such effects are associated with neurobiological mechanisms, particularly the release of endorphins and neurotransmitters such as serotonin and dopamine [21]. In addition, physical activity reinforces a sense of self-efficacy and increases self-esteem [22], regulates sleep [16,18], and improves the physical functioning of cancer patients [10,16,17,23], such as reducing fatigue [10], improving cardiorespiratory capacity, and preserving of muscle mass [16]. Physical activity constitutes an essential role towards enhancing interpersonal interactions and strengthening social support, as it facilitates mutual accountability, provides encouragement, and creates opportunities for sharing cancer-related experiences, which mitigates perceptions of loneliness and social isolation [24,25]. Together, all these factors are fundamental to physical and psychological well-being, facilitating the recovery process from cancer treatment and, therefore, the cancer survivors’ quality of life [17,20,23]. As reported by Sun et al. [19], meta-analysis showed that physical activity can considerably enhance quality of life for breast cancer survivors.

Research indicates that cancer survivors benefit from group-based programs or professional supervised exercise, as this environment provides better quality of life and physical activity outcomes [18,26,27]. Therefore, in recent years, there has been a growing interest in personalized, supervised, and structured physical activity programs for cancer survivors.

Although physical activity levels among the cancer population in general are low, cancer survivors living in rural and remote areas are even less likely to follow the recommendations, contributing to several disparities in cancer care access and poorer overall health [28,29]. These disparities are the result of several factors, including limited access to structured rehabilitation and physical activity programs and interventions, lower access to healthcare and cancer support services, geographic isolation, and lower levels of literacy [28,29,30]. In Portugal, there is a growing interest in studying physical activity and exercise among survivors of different types of cancer, especially breast cancer, conducted in clinical trials or quasi-experimental designs, which feature supervised exercise programs that last for several weeks [31,32]. Some key limitations identified include poor adherence to the programs, the lack of a control group, the absence of randomization, and limited sample sizes [31,32]. However, a large disparity persists, since there have been limited studies involving cancer survivors living in rural regions and the interior of the country, highlighting that there is a gap in the access to supervised physical exercise programs.

The present study aims to assess the mental health of cancer survivors, according to levels of depression and anxiety, and their relationship with engagement in physical activity, as well as the effect on quality of life.

## 2. Materials and Methods

### 2.1. Study Design

The quantitative approach was employed as the research methodology to achieve the proposed objectives of this study. Therefore, the present study follows a cross-sectional, descriptive, and correlational design.

### 2.2. Instruments

The sociodemographic and clinical questionnaire included several sections, including sex, age, marital status, socioeconomic status, academic qualifications, current professional situation, period of current diagnosis, current oncological diagnosis, past oncological diagnosis, cancer stage, cancer recurrence, disease stage, metastases or any tumor type than the primary one, treatments conducted, to be carried out, and the date of the last treatment. The questionnaire about physical activity practice was designed to collect specific data related to activity practice, such as the type and frequency of physical activity practiced. When the practice began, the participant’s own initiative to seek information about its benefits, whether it was recommended, participation in any physical activity programs targeted at cancer survivors, among other questions.

Patient Health Questionnaire (PHQ-9) [33] is a self-report diagnostic instrument from the Primary Care Evaluation of Mental Disorders for depression and other common mental disorders. The measure consists of nine items with a 4-point Likert scale ranging from 0 (“not at all”) to 3 (“nearly every day”). In terms of measuring severity, the score can vary between 0 and 27, of which high scores suggest more severe depression [33,34]. In Portugal, evidence of validation has been found in women with breast cancer, with a good internal consistency (Cronbach’s alpha = 0.86) [34]. In this study, it showed good consistency (Cronbach’s alpha = 0.87).

Generalized Anxiety Disorder (GAD-7) [35,36] consists of seven items that measure the participants’ state of anxiety over the last two weeks, using a 4-point Likert scale, from 0 (“not at all”) to 3 (“nearly every day”) [35,37]. In the present research, the GAD-7 showed excellent internal consistency of the seven items (Cronbach’s alpha = 0.90), while the original validation study for the Portuguese population revealed good internal consistency (Cronbach’s alpha = 0.88) [37].

European Organization for Research and Treatment of Cancer Quality of Life Questionnaire Core-30 (EORTC QLQ-C30, version 3) [38] is an instrument consisted of five functional scales (physical, cognitive, emotional, social and role functioning); a global health status and quality of life scale; three symptom scales (fatigue, pain and nausea/vomiting); single items for the assessment of contingent symptoms that are commonly reported by cancer patients; and an item relating to financial concerns and difficulties [38]. The answering scale is a 4-point Likert scale, from 1 (“not at all”) to 4 (“very much”), except for the two items in global health and quality of life scale, where a 7-point modified linear analogue scale is used. The scales and single-item scales score a minimum of 0 and a maximum of 100 [38,39]. The psychometric properties of the Portuguese version of this questionnaire were validated by Pais-Ribeiro et al. [39], with appropriate consistency across all the scales. In the current study, the internal consistency of all the questionnaire items was good (Cronbach’s alpha = 0.83).

Godin Leisure-Time Exercise Questionnaire (GLTEQ) [40,41], has been widely applied to cancer patients in various countries [42] to classify them into various physical categories. Involvement in physical activities is assessed by the Leisure Time Index Score, using three items with a closed response format from 0 to 7, which measures the frequency per week of time spent (at least 15 min) on light (minimal effort, no sweating), moderate (not exhausting, slight sweating) and vigorous (heart beats fast, sweating) aerobic activities during free time in a typical week [40,41]. Despite the low internal consistency coefficient in the present study (0.32), the GLTEQ demonstrates a reliability coefficient of 0.74 [40].

### 2.3. Procedures

Through a sample collected by the non-probabilistic convenience method, the study included all eligible patients referred to by doctor and undergoing medical follow-up in the oncology consultation of a level II hospital, who agreed to participate in the study. Data collection took place among patients available during the waiting period prior to consultation and/or treatment. The ethical approval was obtained from the Ethics Committee of the same institution (ethical approval no. 17/2024). Additionally, a sample was also collected from the “MAMA_MOVE—Supervised Physical Exercise Program for Breast Cancer Survivors,” developed by the Department of Sports Sciences at the University of Beira Interior. Data collection occurred between June and July 2024. All participants were informed of the study’s objectives, confidentiality, and anonymity principles, as well as the voluntary nature of their participation. Following clarification of any questions or concerns, informed consent was obtained through the signed consent forms. Regarding the questionnaire applications, they were self-administered, with assistance from the researchers provided only when necessary. To minimize potential selection biases, previously defined inclusion and exclusion criteria were applied: individuals aged ≥18 years, living in the Cova da Beira region (interior of Portugal), with a cancer diagnosis (any anatomical location or tumor type), undergoing treatment or post-treatment, were included. Only patients in palliative care were excluded. No formal sample size calculation was performed, all eligible patients during the data collection period were included. It is important to highlight that the present study was conducted in accordance with the ethical principles of the Declaration of Helsinki, the World Organization’s Guidelines, and the European Union regulations concerning research involving human subjects. The study also complied with the Portuguese Law No. 21/2014 on clinical research.

### 2.4. Data Analysis

The data was statistically analyzed with the SPSS version 29 software (IBM Corporation, Armonk, NY, USA). The Kolmogorov–Smirnov test was used to evaluate the data distribution. The results revealed that the sample did not follow a normal distribution. Therefore, non-parametric statistical tests were employed. To achieve the first objective, the statistical analysis began with descriptive statistics to describe the sample. Characterization of the sociodemographic, clinic, physical activity questionnaires, as well as the data from the instruments, was conducted using frequencies (*n*), percentages (%), minimum and maximum values, mean (M) and standard deviation (SD). Next, the internal consistency of the instruments in the present study was analyzed by calculating the Cronbach’s alpha. Moreover, the correlation between the variables under study was analyzed using Spearman’s non-parametric correlation coefficient. A multiple linear regression was used to determine whether physical activity improves the relationship between depression, anxiety and quality of life. That is, physical activity scores as the dependent variable and anxiety, depression, and quality of life as independent variables. Therefore, levels of physical activity, depression and anxiety were included in the same model to assess whether higher levels of physical activity mitigate the negative impact of depression and anxiety on the quality of life of cancer survivors.

The results were considered statistically significant when the *p*-value did not exceed a significant level of 5%.

## 3. Results

### 3.1. Sociodemographic, Clinical, Physical Activity, and Psychological Characteristics of the Sample

This study included participants aged 18 or older, living in inland of Portugal, who have been diagnosed with cancer (regardless of anatomical location, tumor type, or stage of treatment and post-treatment). Individuals undergoing palliative care were excluded.

The sample consisted of 55 participants, with a predominance of females (69.1%), of whom 31 (56.4%) were married and 21 (38.2%) reported being in the low-middle socioeconomic status. Ages ranged from 40 to 84 (62.27 ± 11.91). Regarding educational qualifications, the majority had a 4th year degree and a 12th year degree, both with 25.5%, and 25 (45.5%) participants were retired. In terms of clinical characteristics, 26 (47.3%) participants reported having breast cancer, diagnosed a year or less ago (41.8%) or two to five years ago (40.0%) and with stage II of cancer (18.2%). At the time of data collection, most participants were in remission from the disease (52.7%) (Appendix A.1). As part of the preliminary validation process for the GLTEQ instrument, an exploratory analysis was performed to assess potential differences among different types of cancer. Although no significant differences were identified (*p* = 0.063), it was observed that the mean score for breast cancer (26.62 ± 15.38) was higher than the mean score for the other type of cancer. No differences were calculated between types of cancer and the psychopathological symptoms variables.

In relation to exercise-related characteristics, 46 cancer survivors (83.6%) reported engaging in physical activity. The most commonly practiced type included outdoor activities (e.g., walking, running, cycling) (43.6%) with a frequency ranging between once to twice a week (25.5%), three to four times a week (29.1%), every day (21.8%) and occasionally (7.3%). In addition, 17 participants (30.9%) reported exercising mostly alone, 14 (25.5%) in group settings, 16 (29.1%) in both modalities, and eight participants (14.5%) either did not respond or stated that the question did not apply. When asked if they were part of any physical activity program specifically designed for cancer survivors, 17 individuals (30.9%) answered “yes”, 35 (63.6%) answered “no”. Furthermore, 29 participants (52.7%) reported not being supervised by a professional (e.g., personal trainer, physiotherapist), and, finally, 44 (80.0%) stated feeling benefits associated with physical activity (Appendix A.2).

Another data to consider is that 43 participants (78.2%) had no previous psychological counselling, and 16 (29.1%) answered having been diagnosed with a psychological or psychiatric condition (Appendix A.3).

### 3.2. Descriptive Statistical Analysis of the Instrument’s Items

A descriptive statistical analysis was performed on the three items that constitute the instrument that measures the practice of physical activity (GLTEQ). The total variation in the GLTEQ ranged between a minimum of zero to a maximum of 56, with a mean value of 19.76 ± 16.20. The three items’ mean score ranged from 0.07 and 3.40, with the highest mean score obtained in item 3 “Mild/light exercise” (3.40 ± 4.63). Each item’s descriptive statistical analysis is shown in Table 1.

In terms of the total GLTEQ classifications (see Table 2), 22 (40%) participants were classified as “Insufficiently active/sedentary”, 15 (27.3%) participants as “Moderately active”, and 18 (32.7%) participants as “Active”. However, although nearly half of the sample was classified as “Insufficiently active/sedentary”, 33 (60%) individuals exhibited non-sedentary scores, indicating their engagement in physical activity.

According to the data presented in Table 3, the mean of the GLTEQ score was 19.76 ± 16.20 (95% CI: 15.38 to 24.14), stating that the participants are considered “Moderately active”. The mean obtained on the PHQ-9 was 6.75 ± 5.84 (95% CI: 5.17 to 8.33), which suggests that the cancer survivors in this sample have mild depressive severity. Similarly, the mean score for the GAD-7 was 4.65 ± 4.72 (95% CI: 3.38 to 5.93), indicating that the participants had normal to average levels of anxiety. As for the EORTC QLQ-C30, the cancer survivors in the sample had an average score of 58.79 ± 22.01 (95% CI: 52.84 to 64.74), imply that the participants viewed their quality of life as moderate.

### 3.3. Relationship Between Physical Activity, Psychopathological Symptoms, and Quality of Life

The relation between physical activity, psychopathological symptoms and quality of life (see Table 4) showed a positive and weak association with physical activity and quality of life (r = 0.170; *p* = 0.216), indicating that, despite not being statistically significant, as levels of physical activity increase, quality of life tends to improve. Physical activity and depression were negatively correlated (r = −0.189; *p* = 0.166), i.e., people who exercise more tend to have fewer depressive symptoms, although this relationship is not strong or significant. As for the association between physical activity and anxiety, it was negative and weak (r = −0.103; *p* = 0.454), meaning that higher levels of physical activity are associated with a reduction in anxiety symptoms, even if this is a slight and non-significant association.

In contrast, a negative and significant correlation (r = 0.496; *p* < 0.01) was found between quality of life and depression, suggesting that higher levels of quality of life are moderately associated with lower levels of depression. The relationship found between quality of life and anxiety was negative and significant (r = 0.406; *p* < 0.01), i.e., higher levels of quality of life are significantly tied to lower levels of anxiety. However, there was a strong positive correlation (r = 0.749; *p* < 0.01) seen between depression and anxiety, suggesting that these two variables are highly connected.

A series of regressions were conducted for each of these variables, and no significant patterns were found for depression or physical activity. A total of 33.6% of quality of life was explained by depression, anxiety and physical activity (R^2^ = 0.336; F(5,49) = 4.966, *p* < 0.001), with anxiety having a negative effect on quality of life (β = −0.742; t = −2.672; *p* = 0.010). As for the interaction between depression and physical activity, a negative and significant impact on quality of life was found (β = −0.946; t = −3.296; *p* = 0.002). The interaction between anxiety and physical activity was found to have a positive and significant effect on quality of life (β = 0.763; t = 2.590; *p* = 0.013). Thus, the interactions between physical activity and depression, as well as between physical activity and anxiety, were found to be significant, indicating that physical activity may serve as a predictor of the relationship between psychopathological symptoms and quality of life. These results can be found in Table 5.

## 4. Discussion

The present study proposed to describe and assess the practice of physical activity in adult cancer survivors. Additionally, the study aimed to analyze the health of cancer survivors by examining how physical activity relates to psychopathological symptomatology and their impact on quality of life.

In terms of describing the practice of physical activity in leisure time, the values observed can be attributed largely to the small and limited number of participants in the sample and the number of items in the instrument (GLTEQ), as well as the homogeneity of the participants’ physical activity routines (e.g., outdoor activities). A majority (98.4%) reported not practicing “Vigorous exercise” on any day per week. Given that the GLTEQ is a self-administered questionnaire, this fact may be related to the participants’ focus on the examples given in the items rather than their literal meaning. This may have influenced the results, resulting in 40% of participants being classified as “insufficiently active/sedentary”. These results are consistent with previous studies, such as Avancini et al. [43], who, when using the same instrument, reported that less than 10% of patients were identified as “sufficiently active”. Furthermore, the fact that the participants reported not exercising vigorously or moderately a few times a week or not at all, reflected 27.3% and 32.7% of participants being classified as “moderately active” and “active”, respectively. This observation corroborates other studies, such as Smith-Tuchyn et al. [44], who only identified 37% of their participants as “sufficiently active”.

Despite the lack of significance associations between psychological variables and physical activity, those may be influenced by uncontrolled clinical and sociodemographic factors, as well as the limited sample size and low variability in physical activity levels. However, the study found that anxiety has a significant negative influence on the quality of life of cancer survivors. Although depression is not a significant predictor of quality of life, its interaction with physical activity levels indicates that the latter can attenuate the effect of depression. Therefore, physical activity levels can attenuate the negative impact of psychopathological symptoms on quality of life. Furthermore, engaging in physical activity affects psychological symptoms and overall quality of life through a variety of interconnected mechanisms. From a neurobiological perspective, there is an increase in the release of endorphins and neurotransmitters such as serotonin and dopamine, which contribute to the regulation of mood and emotions [21], as well as a reduction in chronic pain [9]. Psychologically, research shows improvements in self-efficacy and self-esteem [22]. Regarding social mechanisms, the reinforcement of social interactions and social support is particularly notable [24,25]. Together, these processes can produce significant outcomes, such as sleep regulation [16,18], decreased fatigue [10], and reduced feelings of loneliness and social isolation [24,25]. In fact, several studies indicate that physical activity plays a crucial role in improving the quality of life of cancer survivors, positively impacting on their mental health [10,19,20] and consequently contributing to reducing the risk of recurrence and mortality in cancer patients [10]. As well as physical activity improving depressive and anxiety symptoms, it is important to note that the opposite can also occur. In other words, anxiety and depression can influence physical performance [45].

Overall, improving the physical and functional capacity of cancer patients through physical activity provides a sense of psychological relief, by decreasing stress, depression, and anxiety [46,47]. However, these effects can be different depending on the type and intensity of physical activity. Furthermore, physical activity is not sufficient to directly eliminate these psychopathological symptoms, but it does help to mitigate their effects on quality of life. A study found that the vigorously active group of cancer patients had higher mental health and perceived health scores, followed by the moderately active and inactive groups [24]. These results indicate that physical activity can be an essential therapeutic intervention to promote quality of life and well-being in cancer patients [24]. These results highlight the importance of promoting physical activity in both the general population and the cancer population, in addition to developing and encouraging prevention and rehabilitation programs for cancer patients. Several studies provide evidence that these programs tend to increase levels of physical activity [48], while also showing positive effects on various aspects of quality of life, such as physical aptitude and social support [24,27].

There are some limitations to the study. Firstly, it should be emphasized that this study, being cross-sectional, does not allow for a temporal sequence of the results. The findings do not allow for the establishment of cause-and-effect relationships, which only identifies associations between the variables studied. Therefore, it is not possible to ensure whether physical activity contributes to a better quality of life and mental health, or whether cancer survivors with better mental health and a higher quality of life are more likely to engage in physical activity. The study’s analysis focused particularly on the variables under investigation, and potential confounding factors (e.g., age, sex, type of cancer) were not controlled for, which may have influenced the associations observed. Also, there is a strong correlation between anxiety and depression, indicating a potential multicollinearity. This relationship makes it difficult to distinguish the specific impacts of each variable, therefore our findings should be interpreted with care. Furthermore, the fact that the sample was obtained by convenience and restricted to a specific group, rather than by probability, prevents the generalization and external validation of its results. Clear criteria for representing the general population are crucial, including the selection of participants from different regions of the country and with more varied clinical characteristics, making it possible to generalize the results to the Portuguese population. The use of a small, aged, and heterogeneous sample made it difficult to provide a more solid analysis of the effects of physical activity and psychopathological symptoms on quality of life. The other limitation of this study lies in the fact that physical activity levels were measured using self-report instruments, without any objective assessment of physical activity levels in cancer survivors (e.g., heart rate monitors and movement sensors). Furter studies with larger, more representative samples and controlling for potential confounders may contribute to improving the findings’ strength and external validity. Furthermore, incorporating more detailed analyses of collinearity may provide clearer insights into the distinct contributions of each variable. Experimental and longitudinal studies are needed to clarify and corroborate the role of physical activity and its relationship to improving quality of life and mental health in cancer survivors. Other psychopathological symptoms, including somatization, could also be explored.

Nevertheless, important results were obtained, which contribute to increasing and consolidating theoretical knowledge on this subject, as well as adding evidence to the subjective assessment of physical activity levels in cancer survivors in Portugal, specifically in the interior region of the country. The results show that cancer patients who practice more physical activity have fewer psychopathological symptoms and higher levels of quality of life. This emphasizes the need to promote healthier lifestyles, including the use of supervised physical activity, as well as the development of health promotion and education. It also highlights the importance of sensitizing and raising awareness among healthcare professionals to recommend and promote physical activity as part of an integrated healthcare with continuous professional support. Therefore, it is essential to have a personalized, idiosyncratic, and informed intervention in the care of cancer survivors, focused on both the importance of physical activity and the prevention of psychopathological symptoms by providing psychological support.

## 5. Conclusions

This study has led to some important conclusions, in particular recognizing that the participants in this study had appropriate levels of physical activity, with a majority categorized as “moderately active” or “active”. The main modality of choice for cancer survivors was outdoor activities, namely walking. The interactions between physical activity and depression, as well as physical activity and anxiety, are predictors of quality of life in the population studied. Therefore, physical activity can contribute to improving the perceived quality of life of cancer survivors, moderating the negative effects of psychopathological symptoms.

In terms of practical implications for clinical care, these findings not only highlight the importance of easing awareness among healthcare professionals but also emphasize their essential role in effectively promoting physical activity in a personalized and integrated way. For that reason, healthcare professionals should evaluate each individual’s preferences and barriers; motivate and provide counseling focused on realistic, gradual and achievable goals; encourage healthy lifestyles; integrate assistance from a multidisciplinary team; and develop rehabilitation and physical activity programs.

## Figures and Tables

**Table 1:** Descriptive statistical analysis of weekly frequency of physical activities, according to GLTEQ (*n* = 55).

Intensity	None *n* (%)	1 Time *n* (%)	2 Times *n* (%)	3 Times *n* (%)	4 Times *n* (%)	5 Times *n* (%)	6 Times *n* (%)	7 Times *n* (%)
Vigorous exercise (with rapid heartbeat)	53 (96.4%)	1 (1.8%)	0 (0%)	1 (1.8%)	0 (0%)	0 (0%)	0 (0%)	0 (0%)
Moderate exercise (not exhausting)	29 (52.7%)	1 (1.8%)	7 (12.7%)	7 (12.7%)	4 (7.3%)	1 (1.8%)	1 (1.8%)	5 (9.1%)
Mild/light exercise (minimal effort)	13 (23.6%)	1 (1.8%)	9 (16.4%)	7 (12.7%)	7 (12.7%)	3 (5.5%)	1 (1.8%)	14 (25.5%)

Abbreviation: *n* = frequency; % = percentage; GLTEQ = Godin Leisure-Time Exercise Questionnaire.

**Table 2:** Descriptive statistical analysis of the GLTEQ classifications (*n* = 55).

GLTEQ Classifications	*n* (%)
Insufficiently active/sedentary	22 (40%)
Moderately active	15 (27.3%)
Active	18 (32.7%)

Abbreviation: *n* = frequency; % = percentage; GLTEQ = Godin Leisure-Time Exercise Questionnaire.

**Table 3:** Descriptive statistical analysis of the GLTEQ, PHQ-9, GAD-7 and EORTC QLQ-C30 scores (*n* = 55).

Scales	*n* (%)	Min.–Max.	Mean ± SD	95% CI
GLTEQ	55 (100%)	0–56	19.76 ± 16.20	[15.38, 24.14]
PHQ-9	55 (100%)	0–27	6.75 ± 5.84	[5.17, 8.33]
GAD-7	55 (100%)	0–18	4.65 ± 4.72	[3.38, 5.93]
EORTC QLQ-C30	55 (100%)	0–100	58.79 ± 22.01	[52.84, 64.74]

Abbreviation: GLTEQ = Godin Leisure-Time Exercise Questionnaire; PHQ-9 = Patient Health Questionnaire; GAD-7 = Generalized Anxiety Disorder; EORTC QLQ-C30 = European Organization for Research and Treatment of Cancer Quality of Life Questionnaire Core-30; *n* = frequency; % = percentage; Min. = minimum; Max. = maximum; SD = standard deviation.

**Table 4:** Spearman Correlation Matrix among Physical Activity, Quality of Life, Depression, and Anxiety (*n* = 55).

Scales	Physical Activity	Quality of Life	Depression	Anxiety
Physical Activity				
Quality of Life	0.170			
Depression	−0.189	−0.496 *		
Anxiety	−0.103	−0.406 *	0.749 *	

Note: * *p* < 0.01.

**Table 5:** Multiple Linear Regression: Depression, Anxiety and Physical Activity as Predictors of Quality of Life (*n* = 55).

Variable	B (Coefficient)	SD	*β* (Standardized Coefficient)	t	*p* (Sig.)	95% CI
(Constant)	61.030	6.311	-	9.671	<0.001 **	[48.348, 73.712]
Depression	1.701	1.005	0.451	1.693	0.097	[−0.318; 3.720]
Anxiety	−3.463	1.296	−0.742	−2.672	0.010 *	[−6.069, −0.858]
Physical Activity	0.462	0.234	0.340	1.978	0.054	[−0.007, 0.932]
Depression x Physical Activity	−0.181	0.055	−0.946	−3.296	0.002 *	[−0.291, −0.070]
Anxiety × Physical Activity	0.165	0.064	0.763	2.590	0.013 *	[0.037, 0.293]

Note: Dependent Variable = Quality of Life; * *p* < 0.05, ** *p* < 0.001; SD = standard deviation; CI = confidence interval.

## Data Availability

The raw data supporting the conclusions of this article will be made available by the authors on request.

## Abbreviations

The following abbreviations are used in this manuscript:

PHQ-9	Patient Health Questionnaire
GAD-7	Generalized Anxiety Disorder
EORTC QLQ-C30, version 3	European Organization for Research and Treatment of Cancer Quality of Life Questionnaire Core-30
GLTEQ	Godin Leisure Time Exercise Questionnaire

## Appendix A

### Appendix A.1

**Table A1:** Sociodemographic and clinical characteristics of the sample (*n* = 55).

		*n* (%)
Sex	Female	38 (69.1%)
	Male	17 (30.9%)
Marital status	Single	5 (9.1%)
	Married	31 (56.4%)
	Civil partnership	6 (10.9%)
	Divorced or separated	8 (14.5%)
	Widowed	5 (9.1%)
Socioeconomic status *	Low	9 (16.4%)
	Low-medium	21 (38.2%)
	Medium	15 (27.3%)
	Medium-high	4 (7.3%)
	High	6 (10.9%)
Academic qualifications	Less than four years of schooling	2 (3.6%)
	4th year complete	14 (25.5%)
	6th year complete	2 (3.6%)
	9th year complete	9 (16.4%)
	12th year complete	14 (25.5%)
	Bachelor’s degree complete	9 (16.4%)
	Master’s degree complete	3 (5.5%)
	Doctoral’s degree complete	2 (3.6%)
Current professional situation	Active (working)	15 (27.3%)
	Medical leave	12 (21.8%)
	Unemployed	2 (3.6%)
	Retired	25 (45.5%)
	Other	1 (1.8%)
Period of current diagnosis	1 year or less	23 (41.8%)
	2 to 5 years	22 (40%)
	6 to 10 years	2 (3.6%)
	More than 10 years	7 (12.7%)
	No reply	1 (1.8%)
Current oncological diagnosis	Colon and rectum	13 (23.6%)
	Stomach	3 (5.5%)
	Breast	26 (47.3%)
	Ovary	1 (1.8%)
	Prostate	3 (5.5%)
	Leukemia	1 (1.8%)
	Lung	7 (12.7%)
	Other	1 (1.8%)
Cancer stage	0 or carcinoma in situ	6 (10.9%)
	I	5 (9.1%)
	II	10 (18.2%)
	III	9 (16.4%)
	IV	3 (5.5%)
	Do not know	22 (40%)
Disease stage	Awaiting treatment	1 (1.8%)
	Under treatment	21 (38.2%)
	Awaiting results	2 (3.6%)
	In remission	29 (52.7%)
	Relapse	1 (1.8%)
	No reply	1 (1.8%)

Note: * The following classification was considered regarding socio-economic status: participants classified as “low” answered “less than 499 euros per month per household”, participants classified as “low-medium” answered “between 500 and 999 euros per month per household”, participants classified as “medium” answered “between 1000 and 1499 euros per month per household”, participants classified as “medium-high” answered “between 1500 and 1999 euros per month per household” and participants classified as “high” answered “more than 2000 euros per month per household”.

### Appendix A.2

**Table A2:** Physical exercise characteristics of the sample (*n* = 55).

		*n* (%)
Practicing physical exercise	Yes	46 (83.6%)
	No	9 (16.4%)
Physical activity and exercise modalities	Outdoor activities (e.g., running, walking, cycling)	24 (43.6%)
	Gym/Fitness (e.g., personal trainer, weight room)	6 (10.9%)
	Physiotherapy (e.g., stretching)	1 (1.8%)
	Group classes (e.g., pilates, yoga)	1 (1.8%)
	Aquatic activities (e.g., swimming, aqua aerobics)	0 (0%)
	Team sports (e.g., football, basketball)	0 (0%)
	Outdoor activities and Gym/Fitness	7 (12.7%)
	Outdoor activities and Physiotherapy	3 (5.5%)
	Outdoor activities and Group classes	1 (1.8%)
	Gym/Fitness and Physiotherapy	1 (1.8%)
	Gym/Fitness and Aquatic activities	1 (1.8%)
	Outdoor activities, Gym/Fitness and Aquatic activities	1 (1.8%)
	Other (e.g., tennis, padel, golf)	0 (0%)
	No	9 (16.4%)
Frequency of physical exercise	Once or twice a week	14 (25.5%)
	Three to four times a week	16 (29.1%)
	Every day	12 (21.8%)
	Occasionally	4 (7.3%)
	I do not exercise at the moment	9 (16.4%)
Recommendation of physical exercise by healthcare professionals	Yes	39 (70.9%)
	No	14 (25.5%)
	No answer/not applicable	2 (3.6%)
Do you mostly physical exercise	Alone	17 (30.9%)
	In a group	14 (25.5%)
	Both	16 (29.1%)
	No answer/not applicable	8 (14.5%)
Participation in a physical exercise program for cancer patients	Yes	17 (30.9%)
	No	35 (63.6%)
	No answer/not applicable	3 (5.5%)
Supervised practice of physical exercise	Yes	22 (40%)
	No	29 (52.7%)
	No answer/not applicable	4 (7.3%)
Feeling the benefits of physical exercise	Yes	44 (80%)
	No	3 (5.5%)
	No answer/not applicable	8 (14.5%)

### Appendix A.3

**Table A3:** Clinical psychological counselling characteristics of the sample (*n* = 55).

		*n* (%)
Psychological counselling	Yes	12 (21.8%)
	No	43 (78.2%)
Diagnosis of a psychological or psychiatric illness	Yes	16 (29.1%)
	No	39 (70.9%)
